# Differentiating *Thamnocalamus* Munro from *Fargesia* Franchet *emend*. Yi (Bambusoideae, Poaceae): novel evidence from morphological and neural-network analyses

**DOI:** 10.1038/s41598-017-04613-9

**Published:** 2017-06-23

**Authors:** Shiliang Liu, Rongjie Yang, Jun Yang, Tongpei Yi, Huixing Song, Mingyan Jiang, Durgesh K. Tripathi, Mingdong Ma, Qibing Chen

**Affiliations:** 1 College of Landscape Architecture, Sichuan Agricultural University, Chengdu, Sichuan China; 2 College of Agriculture, Food and Natural Resources, University of Missouri, Columbia, MO USA; 3 Centre for Medical Diagnostic and Research, Motilal Nehru national Institute of Technology, Allahabad, India

## Abstract

*Fargesia* Franchet *emend*. Yi is closely allied with *Thamnocalamus* Munro but differs in many major morphological characteristics. Based on traditional morphological characters, it is difficult to differentiate these two genera. The current study measured 19 species in these two genera to determine whether variations in 12 categories of major characters are continuous. In addition, a self-organizing map (SOM) and cluster analysis were used together to reveal whether the known species of *Fargesia* represent discontinuous sampling of *Thamnocalamus*. The results show that 46 morphological characteristics exhibited high variation at the generic and species levels. In addition, the cluster analysis showed that 32 morphological characteristics of *Thamnocalamus* and *Fargesia* were divided between two species and well separated from the outgroup. Additionally, significant differences (*P* < 0.01) were observed in the reproductive structures between these two genera. The unrooted dendrogram, which was based on the SOM neural network, shows the same results as the cluster analysis of morphological characteristics. These data indicate that *Fargesia* is not a result of discontinuous sampling of *Thamnocalamus*; thus, *Fargesia* should not be treated as a synonym for *Thamnocalamus*.

## Introduction

Bambusoideae (bamboo) represents one of 12 recognized sub-families of perennial grasses (Poaceae, formerly known as Gramineae) and is widely found from sea level to high plateaus worldwide (ca. 4000 m.a.s.l. in the Himalayas), primarily in forest and grassland habitats, except for in Europe and Antarctica (see Figure S1)^1–3^. Bamboo is also one of the most valuable plants; it is used for many goods and services^4, 5^. However, information on the distribution, ecology and intraspecific variation of bamboo is inadequate, and its biodiversity in a broader sense remains largely unexplored^3, 6^. Additionally, the taxonomic characteristics that can be used to classify or group bamboo are confusing, and estimates of the total diversity have described 1,480 species in 115 genera (ca. 48 genera and nearly 500 species in China)^2^. Traditional taxonomy has concentrated principally on morphological features, such as the culm and culm-sheath, complex branching, and the rhizome/root system^7, 8^. However, because herbarium specimens are often collected from limited locations and represent a limited number of plants or only parts of a pant, the continuity of variation in characteristics within a species cannot be specifically quantified^9^. For example, numerical taxonomy^10^, one of the traditional taxonomy systems, addresses grouping using numerical methods with taxonomic units based on their character states. In recent years, many authors have treated numerical taxonomy and phenetics as being synonymous^5, 11, 12^ because phenetics, along with cladistics, originated from numerical taxonomy as a way to describe taxonomic relationships based on the patterns of overall similarities and the estimated evolutionary history of the taxa^10^. Although numerical taxonomy seems to be to be effective for species-level classification of woody bamboos, it is very difficult to apply at the genus level^13^, especially among closely related genera, e.g., *Fargesia* Franchet *emend*. Yi*, Thamnocalamus* Munro and *Yushania* Keng f., of woody bamboos^14^. Infrequent flowering events (flowering cycles of up to 120 years)^1^ or flowering only once before culm death, which is one of the special traits in bamboo, restricts the opportunity to gather fresh reproductive material^8, 15, 16^. In addition, inconsistent results among taxonomists usually stem from different interpretations of the characteristics, and confusion with regard to terminology^17, 18^ might result in a virtual species being defined as different species.

To address this issue, advances in modern taxonomic tools have been combined with traditional methods to achieve a more objective classification of bamboo. Recently, DNA sequence data have become available to help review classification systems, such as internal transcribed spacer (ITS) and partial granule-bound starch synthase I gene (GBSSI) sequences^19–21^, restriction site–associated DNA (RAD) and plastid DNA sequences (e.g., *trn*L-F)^22–24^, and random amplified polymorphic DNA (RAPD) and next generation sequencing (NGS) technologies^11, 15^. However, biological data are considered difficult to analyze because numerous biotic/abiotic components are involved in growth processes at all hierarchical levels of life^5, 25^. The components are related not only within but also between the hierarchical levels, eventually leading to trans-disciplinary holism. Therefore, exploring an approach that can convert complex non-linear statistical relationships between high-dimensional data items into simple geometric relationships would be helpful in accurately and effectively analyzing ecological data. In recent years, the development of biologically inspired machine intelligence (BIMI), including supervised and unsupervised learning methods and artificial neural networks (ANNs), has provided convenient tools to understand the ecological and physiological functions of living systems^5, 26^. Among these tools, a self-organizing map (SOM)^27^ is widely utilized because of its similarities to biological nervous systems, its simplicity, and the wide variety of problems to which it might be applied^28^. Lin & Chen^29^ demonstrated that using a SOM is a superior clustering technique and that its relative advantage over conventional techniques increases with higher levels of relative cluster dispersion in the data; it even performed better than seven other hierarchical clustering methods. SOMs have been used extensively for the extraction and clustering of various ecological data, including community classification evaluations^30^, water-quality assessments^31^, and population and community predictions^26, 32^. The Kohonen neural network (or SOM network) can project high-dimensional input space on low-dimensional topologies (Figure S2), thereby overcoming various traditional problems with conventional statistical and operational research techniques^27, 28^. Moreover, many researchers have demonstrated that using a SOM is a superior clustering technique that performs better than other hierarchical clustering methods^25, 28, 32^. These advantages, coupled with the unsupervised nature of the learning algorithm^27^, have made SOMs an attractive and interesting alternative for resolving confusion in plant taxonomy.

Bamboo is a versatile and important component of the ecology, culture, and economic livelihood of people in tropical countries, particularly in the Asia-Pacific region^28, 33^. Bamboo forests are significant for biodiversity, and they provide food and shelter for large animals and birds, soil organisms, insects, and the other plants that compose the bamboo forest ecosystem^34^. The most famous of these is certainly the critically endangered giant panda (*Ailuropoda melanoleuca*), whose distribution is mainly determined by the distribution of *Fargesia* species (e.g., *Fargesia qinlingensis* and *Fargesia robusta*)^35, 36^. *Fargesia* Franchet *emend*. Yi, as part of the *Thamnocalamus* group^37^, comprises approximately 90 species distributed in alpine areas in southwest China, Vietnam, and the adjacent Himalayas^2, 38^. In China, there are 78 species, and 61 of them were recognized and published without a description of their reproductive organs, and many of them are narrowly distributed species^9, 38^. Guo & Li^19^ and Li^39^ divided the *Arundinariinae* into two generic complexes (i.e., *Arundinaria* and *Thamnocalamus* groups) based on the differentiation of rhizome types and leaf anatomy. The *Thamnocalamus* group has pachymorph rhizomes and appears to have large microhairs and dumbbell-shaped silica bodies, although certain species lack fusoid cells^7^. This group consists of *Thamnocalamus* Munro, *Fargesia* Franchet (including *Borinda* Stapleton and *Sinarundinaria* Nakai)^40, 41^ and *Yushania* Keng f. and is closely related to *Ampelocalamus* Chen, Wen et Sheng, *Chimonocalamus* Hsueh et Yi, *Drepanostachyum* Keng f., *Himalayacalamus* Keng f. and *Gaoligongshania* Li, Hsueh et Xia^19, 37, 42^. Although the classification and delimitation of the genera in this group is highly controversial, it remains critical to the systematics of temperate bamboos. Some studies have suggested that *Fargesia* and *Yushania* should be treated as synonyms for the two genera *Thamnocalamus* (with bracteate racemiform synflorescences) and *Sinarundinaria* (with open panicles), respectively^43^. In addition, Soderstrom & Calderon^7^ were the first to propose merging *Fargesia* with *Thamnocalamus* based on their simple, open, semelauctant synflorescence without enclosing spathes, and this classification was also supported by other taxonomists such as Guo *et al*.^37^, Wang & Ye^40^ and Chao & Tang^44^. However, Li *et al*.^45^ believed that *Fargesia* and *Yushania* are comparatively large genera within Arundinarieae with ca. 90 and 80 species, respectively. Species in these two genera are mostly distributed above 2000 m, with some taxa even found at 4000 m, and have pachymorph rhizomes, many branches per node (one to five branches for some Yushania), semelauctant inflorescence and three stamens^21^. In addition, *Fargesia* species have short-necked rhizomes usually with unicaespitose culms and foliage-like bracts under the inflorescence, while *Yushania* species have long-necked rhizomes with diffuse culms and small and reduced bracts under the inflorescence^13^. Therefore, Li^39^ designated *Fargesi*a (*Borinda*, *Sinarundinaria*), *Thamnocalamus* and *Yushania* as the *Thamnocalamus* group and *Ampelocalamus*, *Chimonocalamus*, *Drepanostachyum* (or *Himalayacalamus*) and *Gaoligongshania* as its close allies mainly based on their pachymorph rhizomes and features of their leaf anatomy. One explanation is that attention was paid only to the *Thamnocalamus* group and its allies and that these taxa were resolved as a monophyletic group based on GBSSI and ITS sequences^19, 20^. With more genera in Arundinarieae involved and an analysis of plastid regions^46^, the *Thamnocalamus* group and its allies were shown to be polyphyletic or paraphyletic. Interestingly, Yang *et al*.^23^ confirmed that alpine *Bashania*, *Chimonocalamus*, *Thamnocalamus*, and species currently placed in *Fargesia* and *Yushania* formed a clade based on LEAFY [which is a master regulator orchestrating the whole floral network, and exists across all land plants^47^] and combined their nuclear phylogenies. In addition, based on the original text descriptions and illustrations of a flowering branch with respect to the collections of *Thamnocalamus* species in general, Yi^41^ and Yi *et al*.^48^ noted that the *Thamnocalamus* inflorescences are lateral, whereas *Fargesia* inflorescences are terminal, thereby indicating that *Fargesia* should not be merged with *Thamnocalamus*. Thus, the establishment of a well-defined and unambiguous classification of bamboos requires the adoption of a novel approach.

To verify this issue, specimens of *Thamnocalamus* and *Fargesia* from a total of 19 species, which essentially cover the total distribution of the two genera, were collected. The 46 morphological characteristics for 66 specimens of the two genera were measured and analyzed to determine whether variations in these characteristics are continuous. Furthermore, to improve our understanding of the interspecific relationships of vegetative morphologic characteristics, a SOM, principal component analysis (PCA), and hierarchical cluster analysis (HCA) were used simultaneously for the first time. The SOM technique represents one of the most reliable tools in ecological systematic analyses^26, 28, 32^. Thus, in this work, we attempted to integrate morphological data with SOM-network data to assess differences in the patterns of variation in the characteristics between *Thamnocalamus* and *Fargesia*.

## Results and Discussion

### Morphological and taxonomic comparison of *Thamnocalamus* and *Fargesia*

In this study, the Kruskal–Wallis analysis showed that the values for WOL (presence/absence of leaf sheath), IGF (genuine/false inflorescence), PWOC (two cristae present/absent on palea), PAS (apex split into two in palea), LN (lodicule number), LMH (hairs on margins present/absent in lodicule), SUN (stamen number), and GWOH (hairs present/absent in gynoecium) did not differ among the species studied, which indicates that these characteristics cannot be used to differentiate the species belonging the *Thamnocalamus* and *Fargesia* at *P* < 0.05 (Tables 1 and 2). Among the significant characteristics, 16 morphological characteristics [i.e., TUS (type of underground stem), LSN (number of leaf sheaths), ES (extent of expansion around the spathe in leaf sheath), IB/IC (botryose/conical inflorescences), IC/IS (compact/squarrose inflorescences), IL/IT (terminal/lateral inflorescences), IWOB (bracts present/absent in inflorescences), SN (spikelet number), SFN (number of florets in spikelet), SFC (color of florets in spikelet), SPH (hairs on pedicel present/absent in spikelet), SPB (presence/absence of bracts at the base of pedicel in spikelet), LS (lemmashape), LNN (number of nerves in lemma), PBH (presence/absence of hairs between cristae in lemma), AAC (color of anther in lemma), and GSTN (number of stigmas in gynoecium)] could be differentiated at *P* < 0.01, and ILG (inflorescence length), SLG (spikelet length), LM (lemma texture), and LSSN (relative size of lodicule) could be differentiated at *P* < 0.05 (Tables 2 and 3). Any grouping based on vegetative characteristics should account for parallel trends in inflorescence structure^37^, and many agrostologists have indicated that inflorescences with 6 stamens, such as *Bambusa* Retz. corr. Schreber, are more derived, whereas those with 3 stamens, such *Thamnocalamus* or *Fargesia*, are ancestral^49^. Accordingly, most grasses having 3 stamens^33, 50^ and inflorescences with bracts originated via a reduction of bractless panicles to support vegetative bract-bearing axes. However, according to our field observations, the inflorescence characteristics of representatives of the genera in the *Thamnocalamus* group (see Table 4), including the trend of reduced bracts from *Thamnocalamus* to *Fargesia* and even *Drepanostachyum*, are distinct. This finding is accompanied by a reduction in the presence of bud-like structures in the axes of glumes, an overall expansion from compressed to open inflorescences, and the occurrence of specialized features, such as fasciculation and pulvini, which are more typical of non-bambusoid grass inflorescences^36, 51^. Additionally, in the specimen analysis, inflorescence characteristics (e.g., IB/IC, IC/IS, IL/IT, and IWOB in Table 2) were used to differentiate between the genera at a statistically significant level (*P* < 0.01), indicating that the main differences are the number of spathes and the growth point of the inflorescence. Consistent with these observations, Yi^41^ amended *Fargesia* greatly, adding species without enlarged spathe-like structures and bracteate inflorescences on the basis of shorter rhizome necks. Similarly, Guo *et al*.^37^ and Li *et al*.^45^ clearly discriminated two genera: *Thamnocalamus*, with inflorescence panicles consisting of racemes, with each subtended by a spathe; and *Fargesia*, with terminal inflorescences subtended by several enlarged or not enlarged spathes. We also believe that there is a clear evolutionary trend in spathe size from large to small among *Fargesia* and related genera, which provides strong evidence for the discrimination of species. The trend in spathe size is also correlated with other characteristics [e.g., nutritional (TUS) and reproductive (SN, SFN, and SFC) features], as shown in Table 2 and supported by Guo & Li^19^ and Stapleton^50^. In contrast, a totally different opinion was held by Wang & Ye^40^, who accepted *Fargesia* and *Yushania* as genera in addition to *Thamnocalamus*, with *Sinarundinaria* as a synonym for *Fargesia*, while Chao & Tang^44^ treated *Fargesia* as a synonym for *Thamnocalamus* based on whether the inflorescence was subtended by one to several enlarged spathes. Stapleton^52^ supported the opinion of Wang & Ye^40^ and created a new genus, *Borinda*, which was somewhat intermediate between *Fargesia* and *Yushania*. Thus, some taxonomists have suggested the “very widest” *Arundinaria* Michaux to accommodate the members of the *Thamnocalamus* group from Sri Lanka due to the confusion surrounding these genera.

**Table 1:** Plant material used in the study.

Species	Acronym	Location	No. of individuals	No. of samples	Altitude (m)	Longitude (E)	Latitude (N)
*Fargesia adpressa* T.P. Yi	F.a	Mianning, Liangshan, Sichuan, China	3	16	2360–2700	102.17	28.57
*Fargesia brevissima* T.P. Yi	F.ba	Hongchi, Wuxi, Chongqing, China	3	24	2300–2750	109.07	31.54
*Fargesia denudata* T.P. Yi	F.d	Beichuan, Mianyang, Sichuan, China	3	18	3500–3600	104.81	31.70
*Fargesia dracocephala* T.P. Yi	F.dr	Guandu, Wanyuan, Dazhou, Sichuan, China	3	30	1500–2200	108.06	32.15
*Fargesia ferax* (Keng f.) T.P. Yi	F.f	Kangding, Ganzi, Sichuan, China	3	28	1700–2800	101.95	30.13
*Fargesia fungosa* T.P. Yi	F.fu	Baiji, Weixi, Diqing, Yunnan, China	3	31	1800–2700	99.08	27.36
*Fargesia grossa* T.P. Yi	F.g	Cuona, Shannan, Tibet, China	3	19	2300–3000	91.94	28.21
*Fargesia hsuchiana* T.P. Yi	F.h	Shuiping, Jinping, Honghe, Yunnan, China	3	32	1950–2100	103.24	22.85
*Fargesia lincangensis* T.P. Yi	F.l	Daxueshan, Yongde, Lincang, Yunnan, China	3	21	3300–3400	99.74	24.02
*Fargesia mairei* (Hack. ex Hand.-Mazz) T.P. Yi	F.m	Baidiao, Muli, liangshan, Sichuan, China	3	20	3100–3500	101.46	28.05
*Fargesia melanostachys* (Hand.-Mazz) T.P. Yi	F.me	Baimang snow mountain, Deqin, Yunnan, China	3	22	3300–3400	98.96	28.39
*Fargesia nitida* (Mitford ex Stapf) Keng f.	F.n	Dalu, Jiuzhaigou, Aba, Sichuan, China	3	27	2900–3000	103.67	33.56
*Fargesia ostrina* T.P.Yi	F.b	Taiping,Fengdu, Chongqing, China	3	23	2000–2150	107.73	29.92
*Fargesia pauciflora* (Keng f.) T.P. Yi	F.p	Yaoshang, Qiaojia, Yunnan, China	3	17	2100–2200	103.03	27.08
*Fargesia robusta* T.P. Yi	F.r	Wolong, Wenchuan, Aba, Sichuan, China	3	29	1700–2800	103.19	31.04
*Fargesia spathacea* Franch.	F.sp	Yuanping, Chengkou, Chongqing, China	3	25	1600–2400	108.34	31.92
*Fargesia scabrida* T.P. Yi	F.sc	Beichuan, Mianyang, Sichuan, China	3	26	1450–2520	104.81	31.70
*Thamnocalamus aristatus* E.G. Camus	T.a	Eastern India	1	15	2500–3000	81.51	24.45
*Thamnocalamus unispiculatus* T.P. Yi & J.Y. Shi	T.u	Zhangmu, Nielamu, Shigatse, Tibet, China	14	1~14	2650–3300	85.98	28.00

**Table 2:** Coding of qualitative characteristics into binary or ordered multistate characteristics.

Categories	Characteristics	Acronym	Encoding number				Standard deviation			Kruskal–
			0	1	2	3	TM	FM	All	Wallis Test
Stem	Type of underground stem	TUS	Sympodial-clumping	Sympodial-scattering	—	—	0.00	1.12	1	0.00**
Leaf sheath	Number	LSN	—	—	—	—	0.00	0.00	1	0.00**
	Length relative to inflorescences	CPI	Longer	Shorter	Equal	—	0.49	1.17	1	0.05
	Leaf present or absent	WOL	Absent	Present	—	—	0.00	0.00	0	—
	Extent of expansion around the spathe	ES	Not expanded	Expanded slightly	Expanded	—	0.00	1.01	1	0.00**
Inflorescences	Genuine or false	IGF	False	Genuine	—	—	0.00	0.00	0	—
	Botryose or conical	IB/IC	Botryose	Conical	—	—	0.00	0.94	1	0.00**
	Compact or squarrose	IC/IS	Compact	Squarrose	—	—	0.54	1.06	1	0.00**
	Terminal or lateral	IL/IT	Terminal	Lateral	—	—	0.00	0.00	1	0.00**
	Bracts present or absent	IWOB	Absent	Present	—	—	0.00	0.00	1	0.00**
	Length (mm)	ILG	—	—	—	—	0.90	0.99	1	0.02*
Spikelet	Number	SN	—	—	—	—	0.27	0.81	1	0.00**
	Length (mm)	SLG	—	—	—	—	0.43	1.18	1	0.04*
	Number of florets	SFN	—	—	—	—	0.43	1.07	1	0.00**
	Color of florets	SFC	Light yellow	Light green	Green	—	0.00	0.77	1	0.00**
	Length of rachilla (mm)	SRLG	—	—	—	—	0.38	1.21	1	0.42
	Length of pedicel (mm)	SPLG	—	—	—	—	0.27	1.20	1	0.15
	Hairs on pedicel present or absent	SPH	Absent	Present	—	—	0.00	1.18	1	0.01**
	Bracts at the base of pedicel present or absent	SPB	Absent	Present	—	—	0.00	0.98	1	0.00**
Glume texture	Glume and texture	GM	Papery	Membranous	—	—	1.14	0.92	1	0.76
First glume	Shape	FS	Linear-lanceolate	Ovate-lanceolate	Long/narrow lanceolate	—	0.00	1.29	1	0.20
	Length (mm)	FLG	—	—	—	—	0.49	1.22	1	0.09
	Number of nerves	FNN	—	—	—	—	0.00	1.29	1	0.37
Second glume	Shape	SS	Linear-lanceolate	Ovate-lanceolate	Long/narrow lanceolate	—	0.00	1.30	1	0.21
	Length (mm)	SLG	—	—	—	—	0.45	1.25	1	0.60
	Number of nerves	SNN	—	—	—	—	0.00	1.28	1	0.10
Lemma	Shape	LS	Linear-lanceolate	Ovate-lanceolate	Long/narrow lanceolate	—	0.00	0.85	1	0.00**
	Length (mm)	LLG	—	—	—	—	0.60	1.21	1	0.73
	Number of nerves	LNN	—	—	—	—	0.40	1.09	1	0.00**
	Hairs present or absent	LWOH	Absent	Present	—	—	0.00	1.23	1	0.06
	Texture	LM	Papery	Membranous	—	—	0.00	1.2	1	0.02*
Palea	Length (cm)	PLG	—	—	—	—	0.64	1.15	1	0.15
	Two cristae present or absent	PWOC	Absent	Present	—	—	0.00	0.00	0	—
	Apex split into two	PAS	No	Yes	—	—	0.00	0.00	0	—
	Hairs on cristae present or absent	PCH	Absent	Present	—	—	0.00	1.26	1	0.15
	Hairs between cristae present or absent	PBH	Absent	Present	—	—	0.54	1.06	1	0.00**
Lodicule	Number	LN	—	—	—	—	0.00	0.00	0	—
	Shape	LS	Lanceolate	Triangular	Ovate	—	0.00	1.27	1	0.25
	Relative size	LSSN	Equal	Not equal	—	—	0.00	1.21	1	0.03*
	Hairs on margins present or absent	LMH	Absent	Present	—	—	0.00	0.00	0	—
Stamen	Number	SUN	—	—	—	—	0.00	0.00	0	—
	Color of anther	AAC	Yellow	Brownish yellow	Purple	—	0.00	0.50	1	0.00**
Gynoecium	Shape of ovary	GOS	Elliptic	Ovate	Oblong	—	0.30	1.23	1	0.18
	Hairs present or absent	GWOH	Absent	Present	—	—	0.00	0.00	0	—
	Number of styles	GSN	—	—	—	—	0.00	1.26	1	0.15
	Number of stigmas	GSTN	—	—	—	—	0.46	0.97	1	0.00**

The significance of differences and standard deviations between *Fargesia* and *Thamnocalamus* were calculated with the Kruskal–Wallis test. ** *P* < 0.01, * *P* < 0.05. TM, the mean standard deviation (SD) of *Thamnocalamus* species; FM, the mean SD of *Fargesia* species; All, the SD of *Fargesia* and *Thamnocalamus* species.

**Table 3:** Pearson’s correlation coefficients between the significant discriminatory characteristics as indicated by the Kruskal–Wallis test results in Table 2.

	TUS	LSN	ES	IB/IC	IC/IS	IL/IT	IWOB	SN	SFN	SFC	SPH	SPB	LS	PBH	AAC
LSN	**0.58**														
ES	0.01	**−0.62**													
IB/IC	**−0.60**	**−0.73**	0.31												
IC/IS	**−0.56**	**−0.55**	0.08	**0.66**											
IL/IT	**−0.58**	**−1.00**	**0.62**	**0.73**	**0.55**										
IWOB	**0.58**	**1.00**	**−0.62**	**−0.73**	**−0.55**	**−1.00**									
SN	**0.65**	**0.81**	**−**0.34	**−0.51**	**−0.53**	**−0.81**	**0.81**								
SFN	**−0.53**	**−0.59**	0.14	**0.49**	**0.74**	**0.59**	**−0.59**	**−0.44**							
SFC	0.34	**0.78**	**−0.52**	**−0.47**	**−0.43**	**−0.78**	**0.78**	**0.74**	**−**0.39						
SPH	0.13	**0.50**	**−**0.18	**−**0.21	**−**0.25	**−0.50**	**0.50**	0.31	**−0.55**	0.39					
SPB	0.10	**0.63**	**−0.51**	**−**0.31	**−0.22**	**−0.63**	**0.63**	**0.47**	**−**0.29	**0.66**	0.30				
LS	**−0.51**	**−0.84**	**0.55**	**0.74**	0.54	**0.84**	**−0.84**	**−0.60**	**0.57**	**−0.62**	**−0.47**	**−0.57**			
PBH	**−**0.01	**−0.48**	0.40	0.38	0.32	**0.48**	**−0.48**	**−**0.41	0.11	**−0.67**	**−**0.17	**−0.70**	**0.46**		
AAC	**−**0.39	**−0.91**	**0.59**	**0.58**	**0.46**	**0.91**	**−0.91**	**−0.68**	**0.48**	**−0.69**	**−0.58**	**−0.67**	**0.71**	**0.53**	
GSTN	**0.50**	**0.58**	**−**0.31	**−0.48**	**−0.54**	**−0.58**	**0.58**	**0.61**	**−**0.31	**0.72**	0.30	0.36	**−0.64**	**−0.58**	**−0.46**

Values highlighted in bold are statistically significant at *P* < 0.01(**).

**Table 4:** Feature comparison of the inflorescences of representatives of the genera in the *Thamnocalamus* group.

Genera	Glume buds	Bracts	Fasciculation	Pulvini	Florets
*Thamnocalamus* Munro	Common	Usual	None	No	Many
*Ampelocalamus* Chen, Wen et Sheng	Common	Often	Yes	No	Many
*Fargesia* Franchet	Rare	Few	No	Few	Many
*Yushania* Keng f.	None	None	No	Many	Many
*Drepanostachyum* Keng f.	None	None	Yes	No	2-several
*Himalayacalamus* Keng f.	None	None	Reduced	No	1

In addition, approximately 60% of all studied characteristics showed highly significant correlations (*P* < 0.01; Table 3). Among them, the highest positive correlations were detected between LSN and IWOB (1.00^**^), ACC and LS (0.91^**^), IL/IT and LS (0.84^**^), LSN and SN (0.81^**^), and IWOB and SN (0.81^**^), indicating that the quantifiable characteristics of the leaf sheath and inflorescences are generally dependent on each other. In addition, leaf sheath (LSN) and spikelet (SFC, SPH, and SPB), gynoecium (GSTN) and spikelet (SN and SFC), and palea (PBH) and lemma (LS) characteristics are also positively correlated (*P* < 0.01) with each other. Importantly, differentiations among individuals (i.e., those highly significantly correlated at *P* < 0.01) are also indicated between leaf sheath (ES) and spikelet [SFC (−0.52^**^) and SPB (−0.51^**^)], inflorescence (IB/IC, IC/IS, and IL/IT) and spikelet (SN and SFC; see Table 3), and the stamen (AAC) and spikelet [SN (−0.68^**^), SFC (−0.69^**^), SPH (−0.58^**^), and SPB (−0.67^**^)] characteristics, suggesting that *Fargesia* is actually substantially different from *Thamnocalamus* and presents several more derived morphological characteristics^19, 41, 48^. Thus, recognizing *Fargesia* as a separate genus appears to be reasonable.

### Cluster analysis of vegetative characteristics between *Fargesia* and *Thamnocalamus*

**Figure 1:**
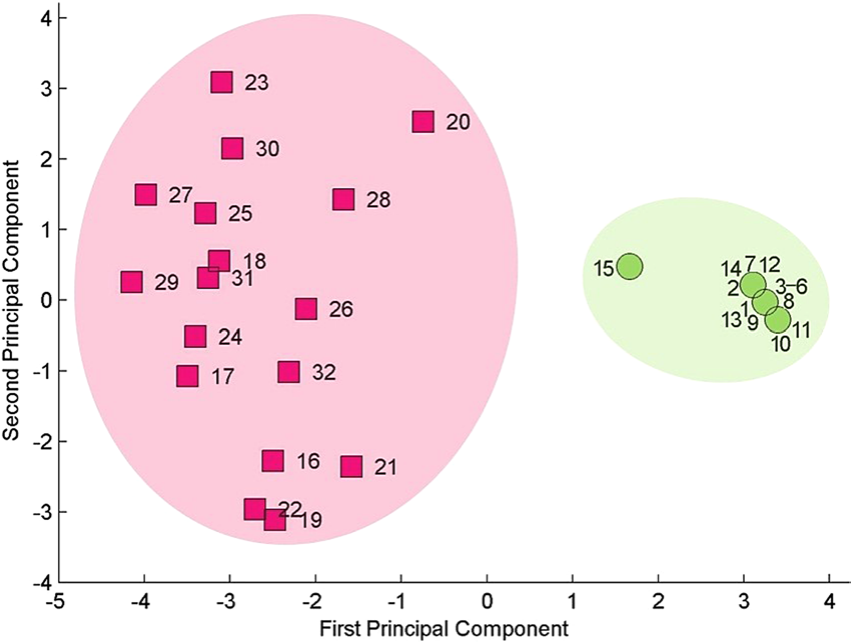
Scatterplot of the scores for the first (PC1) and second (PC2) principal components based on the principal component analysis (PCA). The data were subjected to *z*-score standardization prior to analysis. Red squares represent *Fargesia* species, and green circles represent *Thamnocalamus* species. Please see Table 1 for details.

A PCA was performed to understand how the vegetative characteristics contribute to the definitions of these two genera. A factor analysis showed that the 32 accessions were divided into two distinct groups based on the scatterplot for the two principal component axes (Fig. 1). Evidently, the two groups of accessions comprise the separate genera *Fargesia* (No. 1~15 in Table 1) and *Thamnocalamus* (No. 16~32). Further analysis showed that the 14 specimens of *T. unispiculatus* (No. 1~14) were grouped especially closely along PC1, whereas *T. aristatus* (No. 15) was the most dissimilar specimen in the *Thamnocalamus* group but was separated from the *T. unispiculatus* specimen primarily along PC1. One possible explanation is that *T. aristatus* is a variant of *T. unispiculatus* and that the identification of *T. aristatus* ^52^ is the result of the discontinuous sampling of *T. unispiculatus*. Cases of discontinuous sampling that artificially isolate morphological characters have also been found for bamboo or other taxa^9^. Comparatively, the 17 *Fargesia* species (No. 16~32) showed a maximum distance between absolute values of approximately four units on PC1 and showed greater separation on PC2, with a maximum distance of approximately six units.

**Figure 2:**
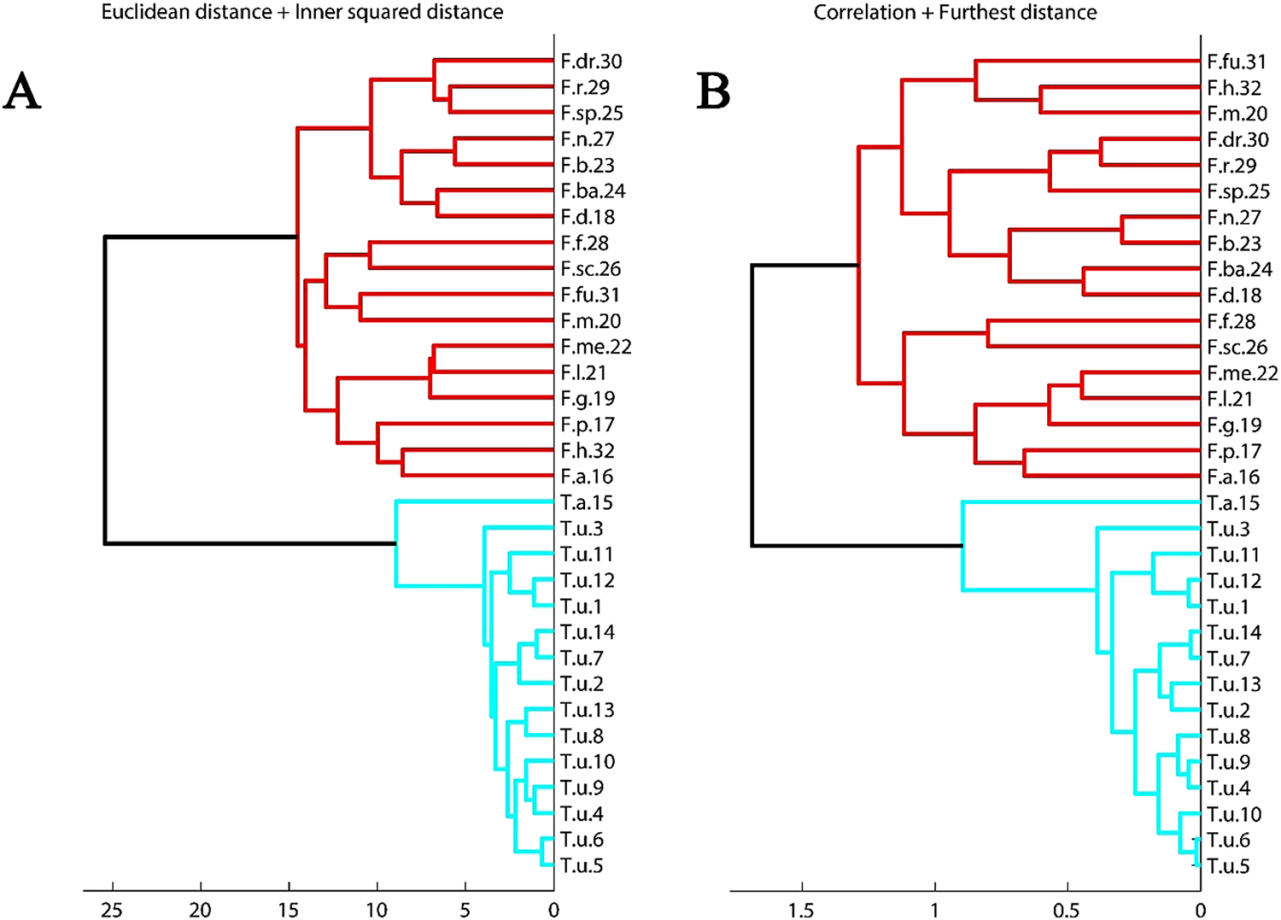
Hierarchical clustering (HC) dendrogram of 32 accessions derived from 46 morphological characteristics. (**A**) Distances between pairs of objects were calculated based on Euclidean distances, and the cluster linkage model utilized the inner squared distance; (**B**) distances between objects (one minus the sample correlation). The cluster linkage model had the greatest distance. In both figures, red lines represent *Fargesia* accessions, and green lines represent *Thamnocalamus* accessions. Please see Table 1 for details.

**Figure 3:**
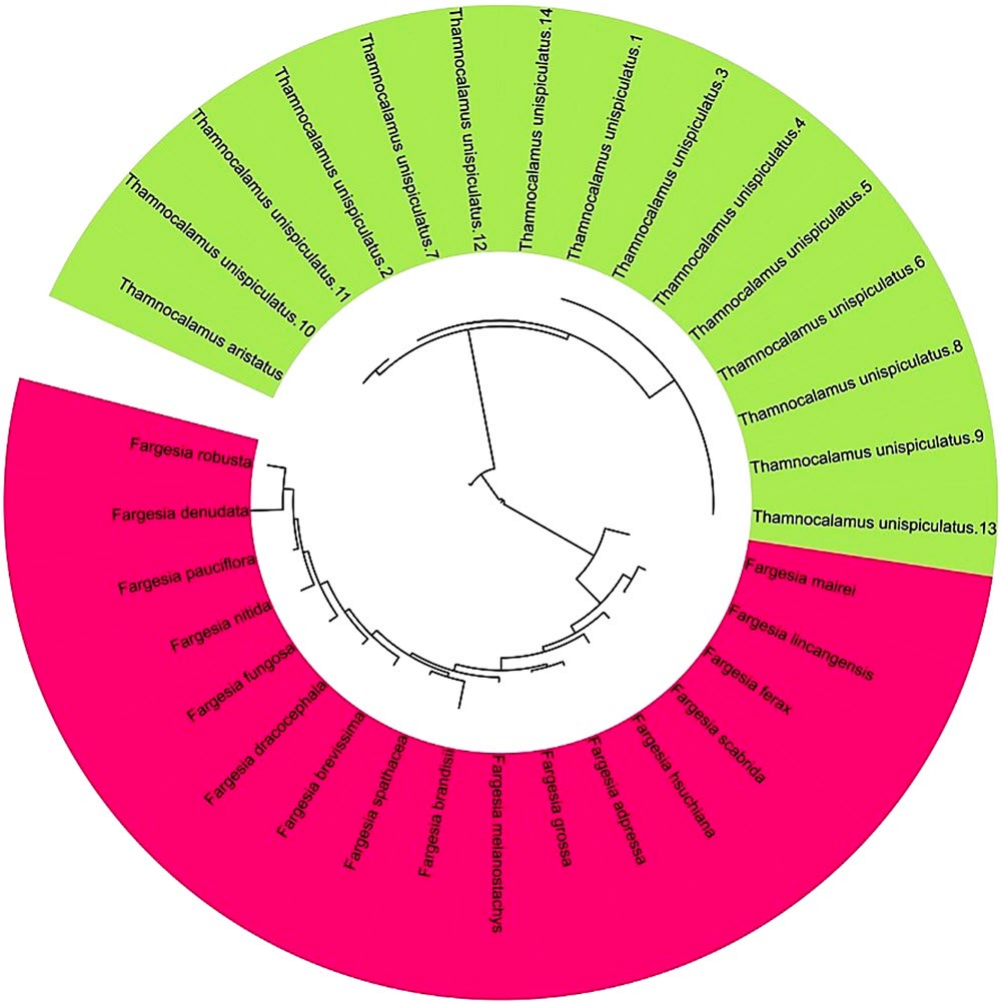
Unrooted dendrogram representing relationships among *Fargesia* and *Thamnocalamus* species estimated with a SOM neural network. For all characters, the mean value was used to construct the dendrogram. Tree distances were calculated with the SOM Neural Network Toolbox for MATLAB software (MathWorks Inc., Natick, MA, USA) and the online tool Interactive Tree Of Life (ITOL; http://itol.embl.de/).

However, in the HCA, the specimens were grouped on the basis of similarities without taking into account information on their class membership^53^. In our analysis, the two most used methods to calculate the distance between pairs of objects in a HCA were applied to analyze the data, and clustering using Euclidean distances grouped the 32 accessions into two main clusters that corresponded to the genera *Fargesia* and *Thamnocalamus* (see Fig. 2A). The maximum intra-cluster distances for the two genera were 14 and 9 Euclidean distance units, whereas a distance of almost 25 Euclidean distance units was observed between these clusters. As shown in Fig. 2, the specimens were obviously divided into two groups, with a large inter-group Euclidean distance. Correlation coefficients between each pair of specimens were calculated as distances and clustered based on a furthest-distance linkage model (Fig. 2B). Both methods had similar cluster results, especially with regard to the consistent division of the 32 accessions into two clusters as *Thamnocalamus* and *Fargesia*. Furthermore, dendrograms generated by the HCA using both Euclidean and city-block distances^54, 55^ and by the SOM neural network also clearly divided the accessions into two distinct clusters corresponding to the two genera, suggesting that no interaction between accessions of *Thamnocalamus* and *Fargesia* was observed and that morphological traits served as effective discriminators of the two genera (Figs 1–3). Therefore, the cluster results also confirmed the hypothesis that *Thamnocalamus* and *Fargesia* should be segregated as separate genera and should not be merged.

### Relationship and discrimination based on the Kohonen neural network

In recent decades, considerable research on bamboo classification has been published; however, systematic studies and critical assessments of taxa are rarely undertaken, which inevitably leads to an increasing number of taxa and little resolution of taxonomic confusion^1, 17, 51^. The issues related to bamboo taxonomy indicate that investigations of bamboo classification should focus on revising taxonomic groups to clarify uncertainties^56^. For example, Zhang *et al*.^9^ committed to integrating the data relating to morphology and ITS regions to assess the patterns of variation in characteristics between two closely allied species (*F. decurvata* and *F. dracocephala*), and the results indicated that *F. dracocephala* should be treated as a synonym of *F. decurvata*. At present, the neural-network approach to recognition and classification is widely used for the discrimination of indistinguishable species^27, 28^, and further investigations should be performed to determine the adaptability and effectiveness of this approach. In this work, the estimated dendrogram, which was based on measured distances^57^ for the morphological characteristics and calculated using a SOM/Kohonen neural network, provided an alternative representation of the relationships among the 32 accessions (Fig. 3). Among them, *T. unispiculatus* was shown to have a very consistent structure; eight specimens had identical phenotypes, whereas a longer branch separated the *T. aristatus* accession from the *T*. *unispiculatus* accessions. Moreover, the longer branch obviously separated the *Fargesia* and *Thamnocalamus* accessions, whereas the branches among the *Fargesia* species were relatively short, suggesting that *Fargesia* should not be treated as a synonym for *Thamnocalamus*. Similarly, the results from the SOM analysis of multiple vegetative characteristics, which has been successful in studies of other species^25, 32^, showed that ten populations were divided into two genera. Generally, a species is a collection of individuals with common characteristics, and defining a species represents the delimitation of the characteristics of the group^8, 9^, with each specimen representing only a reference point for naming. However, if a specimen can be considered to exhibit characteristics that define the species, then continuous variation of characteristics will be presented intermittently, causing difficulties and even errors in classification^7^. Thus, it is essential to investigate as many collections as possible from numerous herbaria, not only from restricted areas but also from entire areas where a taxon may occur.

## Conclusion

As an important constituent of the *Flora*, bamboo is one of the most important plants for humans. However, knowledge of the taxonomic characteristics that can be used to classify or group bamboo species is still rudimentary and confusing. To the best of our knowledge, this is the first detailed report on the discrimination of *Fargesia* Franchet *emend*. Yi from *Thamnocalamus* Munro via an integration of morphological and SOM data to assess the differences in the patterns of variation of characteristics between these two genera. Our main conclusions are as follows: (1) 46 morphological characteristics exhibited considerable variation at the genus and species levels; (2) the PCA and HCA cluster analyses showed that 32 morphological characteristics of *Thamnocalamus* and *Fargesia* were divided among two species and indicated that significant differences (*P* < 0.01) occurred in the floral organs between the two genera; and (3) the unrooted dendrogram based on the SOM neural network representing the relationships among species from the two genera showed the same results as the cluster analysis of the morphological characteristics. These novel findings improve our understanding of the application and role of ANNs (e.g., SOM neural networks) in plant taxonomy. Further studies are required to determine how to merge molecular taxonomy and neural networks and review the classification systems based on morphological traits.

## Materials and Methods

### Plant materials

**Figure 4:**
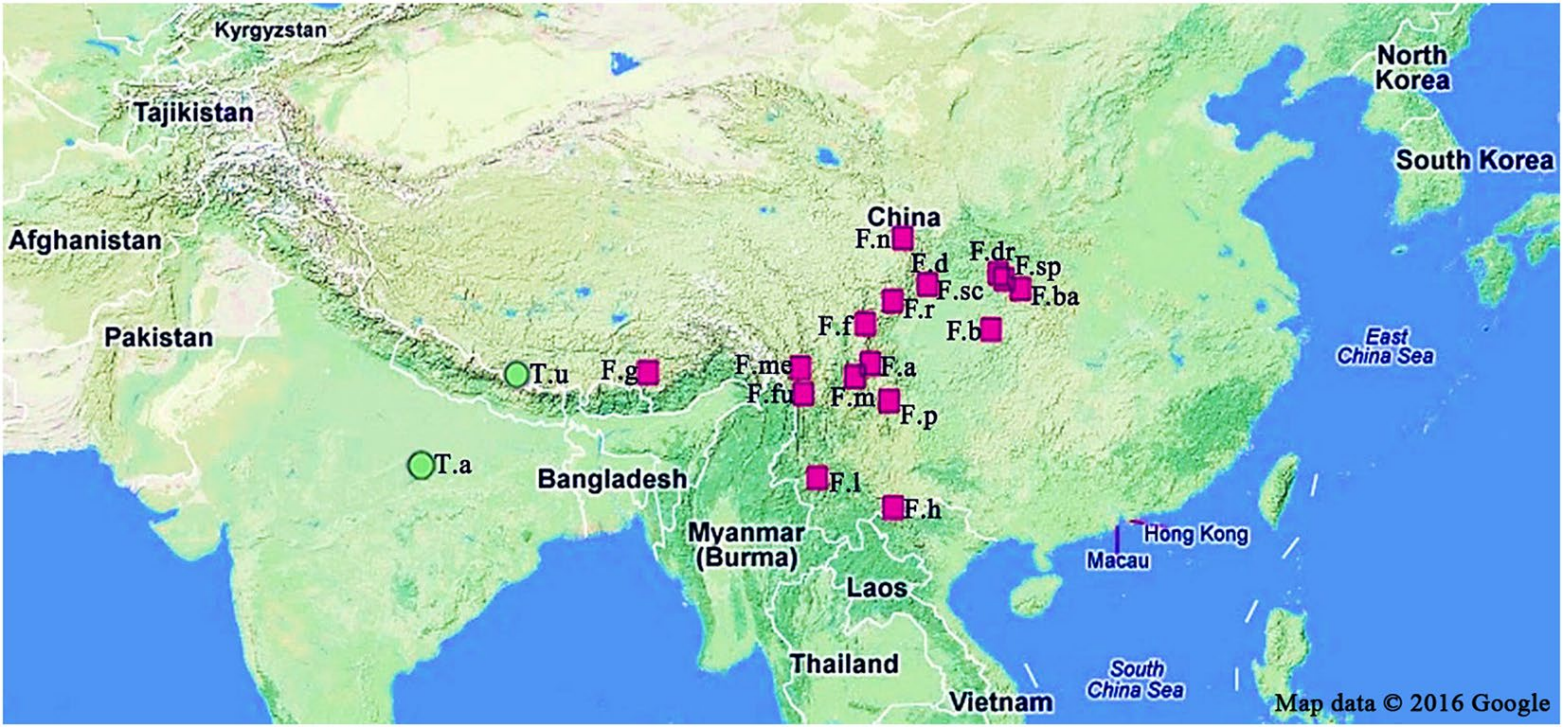
Geographic distribution of accessions of *Fargesia* and *Thamnocalamus* species used in this study. Red squares represent *Fargesia* species, and green circles indicate *Thamnocalamus* species. Please see Table 1 for details. The figure was generated with Adobe^®^ Photoshop^®^ CS3 extend 10.0.1 software (Adobe Systems Inc., San Jose, CA, USA; URL link: https://www.adobe.com/products/photoshop.html ? promoid = KLXLS) based on Google^®^ maps (Google Inc., Mountain View, CA, USA; URL link: https://www.google.com/maps).

Specimens were collected for 19 species, including *Fargesia* Franchet *emend*. Yi (17 species) and *Thamnocalamus* Munro (2 species) (Table 1). Specimens of all the studied species (except for *T. aristatus* E.G., Camus) were collected from the Tibet, Sichuan, Chongqing, and Yunnan provinces in China by Prof. Tongpei Yi from 1975 to 2006. These accessions were deposited in the Campus of Dujiangyan at the Sichuan Agricultural University (SICAU), Sichuan, China. In addition, the accessions of *T. aristatus* were collected in 1960 from eastern India (2500~3000 m.a.s.l.) and obtained from the University of Tokyo (U-Tokyo), Japan. In this work, 15 specimens (15 individuals) from two species (one specimen of *T. aristatus* and 14 specimens of *T. unispiculatus*) in the genera *Thamnocalamus* were studied. Additionally, 17 specimens (54 individuals) in the *Fargesia* genera were studied (see Table 1 and Fig. 4).

### Quantification of morphological characteristics

Based on the *Flora Reipublicae Popularis Sinicae* (FRPS)^38^ and *Flora of China* ^45^, 12 major morphological characteristics and 46 expanded characteristics from a total 19 species were used to identify *Thamnocalamus* and *Fargesia*: (1) type of underground stem [acronym-TUS; see Table 2], (2) leaf sheath (length relative to inflorescences/CPI, LSN, WOL, ES), (3) inflorescences (IGF, IB/IC, IC/IS, IL/IT, IWOB, ILG), (4) spikelet (length of rachilla/SRLG, length of pedicel/SPLG, SN, SLG, SFN, SFC, SPH, SPB), (5) glume texture (GM), (6) first glume (shape/FS, length/FLG, number of nerves/FNN), (7) second glume (shape/SS, number of nerves/SNN, SLG), (8) lemma (LS, LLG, LNN, LWOH, LM), (9) palea (PLG, PWOC, PAS, PCH, PBH), (10) lodicule (LN, LS, LSSN, LMH), (11) stamen (SUN, AAC), and (12) gynoecium [shape of ovary/GOS, number of styles/GSN, GWOH, GSTN] (Tables 1 and 2 and Fig. 4). According to the rule for transforming qualitative data into quantitation indexes (Table S1), we obtained a coding matrix for the studied species (Table S2). In addition, the lengths of the inflorescences, spikelet, rachilla, pedicel, first/second glume, lemma, and palea were measured using a Micrometer Screw-Gauge (Mitutoyo Inc., Kanagawa, Japan).

For the quantitative analysis, all qualitative data for vegetative morphological characteristics were transformed via binary encoding with the following formula^58^:

1 $${x^{\prime} }_{ij=}({x}_{ij}-{\overline{x}}_{i})/{\bar{{s}}}_{j}(i=1,2,\cdots ,n;j=1,2,\cdots ,m)$$

where

2 $${\bar{x}}_{j}=\frac{1}{n}\sum _{i=1}^{n}{x}_{ij},{\bar{s}}_{j}={[\frac{1}{n}\sum _{i=1}^{n}{({x}_{ij}-\overline{{x}_{j}})}^{2}]}^{\frac{1}{2}}(i=1,2,\cdots ,m)$$

To eliminate the impact of dimensional differences between different types of data and enable the use of multivariate analytical techniques, the average value of three individual measurements for each character was calculated and then standardized using the *Z*-score transformation algorithm^59^. Specifically, the raw intensity (I) data for each character were [log_10_] transformed and then used for the calculation of *Z*-scores, which were calculated by subtracting the overall average intensity from the raw intensity data for each character and dividing that result by the standard deviations (*SDs*) of all measured intensities according to the following formula^60^:

3 $$Z \mbox{-} {\rm{score}}=({I}_{C}-{{\rm{MI}}}_{C1}{\ldots }_{C{\rm{n}}})/S{D}_{C1}{\ldots }_{C{\rm{n}}}$$

where *C* is any character of the species, *C*_1_…*C*_n_ represent the aggregate measure of all characteristics, and MI represents the mean intensity. The significance of differences in the mean values of each character between the *Thamnocalamus* and *Fargesia* species were analyzed via the Kruskal–Wallis test^61^.

### Principal component and hierarchical cluster analysis

A principal component analysis (PCA) was applied to the data set after standardization (the mean of the values for each variable was subtracted from each variable value and the result was divided by the standard deviation of the values for each variable). The PCA was performed using the Unscrambler software package (Version 9.7; CAMO Software AS, Oslo, Norway), and it transforms the original, measured variables into new uncorrelated variables called principal components^62^. The first principal component covers as much of the variation in the data as possible. The second principal component is orthogonal to the first and covers as much of the remaining variation as possible (CAMO Software AS, Oslo, Norway). Euclidean distances and city-block distances between accessions were estimated from all recorded characteristics according to Ward Jr.’s method^54^. A HCA was also applied to the standardized data to investigate similarities between different specimens and specimen types (MINITAB, 15.1.1.0, 2007). The HCA calculates the distances (or correlations) between all specimens using a defined metric, such as Euclidean distance or Manhattan distance^18, 53^.

### Self-organizing map (SOM) algorithm

**Figure 5:**
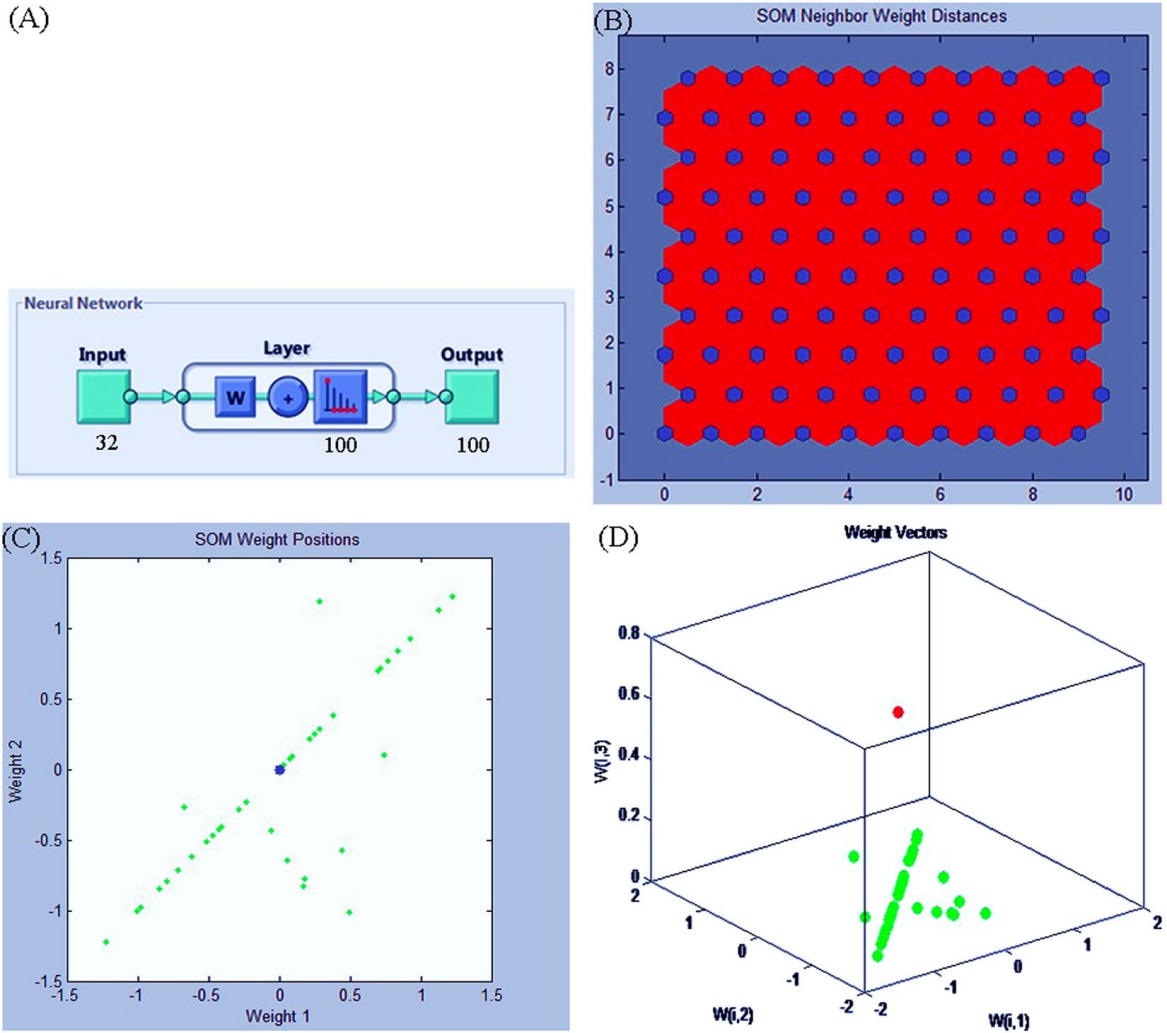
Trained classification structure model (**A**) and weight structure (**B**,**C** and **D**) of the SOM neural network. We converted the 46 morphological characters into normalized vectors of codon usage x(*t*), and 32 accessions were classified by character factors. Symmetrical effects and differences among the samples are more obvious and significant, although off-diagonal weight points are observed.

A SOM is a neural-network algorithm that implements a characteristic nonlinear projection from a high-dimensional space of input signals onto a low-dimensional array of weights^27, 32^. Forty-six morphological characteristics were converted into normalized vectors of codon usage **x**(*t*) (32 accessions were classified by characteristic factors; see Fig. 5). Each component of input vectors was scaled with the following formula so that its mean became 0 and its variance became one:

4 $${x}_{i}^{new}=({x}_{i}^{old}-{o}_{i})/{s}_{i}$$

where *x*_*i*_^old^ is the original value of component *i* of the data vector **x**, O_*i*_ is the mean of values of *x*_*i*_, and *s*_i_ is their *SDs*. The scaling is used to ensure that no component has excessive influence on the learning results due to a greater variance or larger absolute value^63^ (see Supplemental Methods S1).

To avoid dead neurons that might be caused by the random generation of an initial network by the neural network algorithm, the number of training specimens was increased as the network was re-initialized 1000 times. For our analysis, we used the SOM Toolbox (http://www.cis.hut.fi/projects/somtoolbox), a MATLAB-based SOM (MathWorks Inc., Natick, MA, USA), and the interactive Tree Of Life (iTOL; http://itol.embl.de), and a new web-based tool, for the display, manipulation, and annotation of phylogenetic trees^64^.

## Electronic supplementary material

Supplementary information
