# Supplementary material for: Differentiating Thamnocalamus Munro from Fargesia Franchet emend. Yi (Bambusoideae, Poaceae): novel evidence from morphological and neural-network analyses
Source: Sci Rep. 2017 Jun 23;7:4192. doi: 10.1038/s41598-017-04613-9 (PMC5482892; doi:10.1038/s41598-017-04613-9)
Supplement: Supplementary file 1 — Supplementary information [file 41598_2017_4613_MOESM1_ESM.pdf]

Differentiating *Thamnocalamus* Munro from *Fargesia* Franchet *emend*. Yi (Bambusoideae, Poaceae): novel evidence from morphological and neural-network analyses

Shiliang Liu[1, 2], Rongjie Yang[1], Jun Yang[1], Tongpei Yi[1], Huixing Song[1], Mingyan Jiang[1], Durgesh K. Tripathi[3], Mingdong Ma[1] & Qibing Chen[1]

[1] College of Landscape Architecture, Sichuan Agricultural University, Chengdu, Sichuan, China

[2] College of Agriculture, Food and Natural Resources, University of Missouri, Columbia, MO, USA

[3] Centre for Medical Diagnostic and Research, Motilal Nehru national Institute of Technology, Allahabad, India

Correspondence and requests for materials should be addressed to M.D. Ma (email: 610245498@qq.com) and Q.B. Chen (email: cqb@sicau.edu.cn).

SUPPORTING INFORMATION

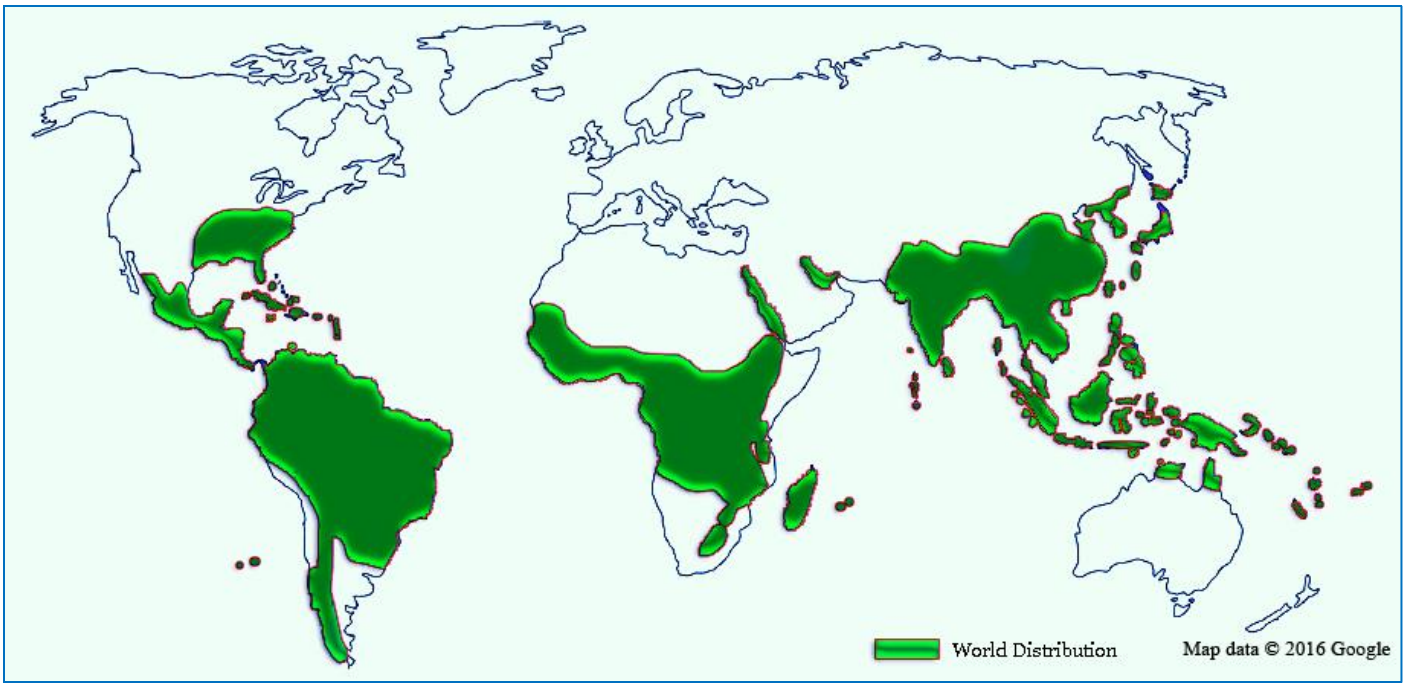

Figure S1. World distribution of bamboo (Poaceae: Bambusoideae). The figure was generated with Adobe® Photoshop® CS3 extend 10.0.1 software (Adobe Systems Inc., San Jose, CA, USA; URL link: https://www.adobe.com/products/photoshop.html?promoid=KLXLS) based on Google® maps (Google Inc., Mountain View, CA, USA; URL link: https://www.google.com/maps).

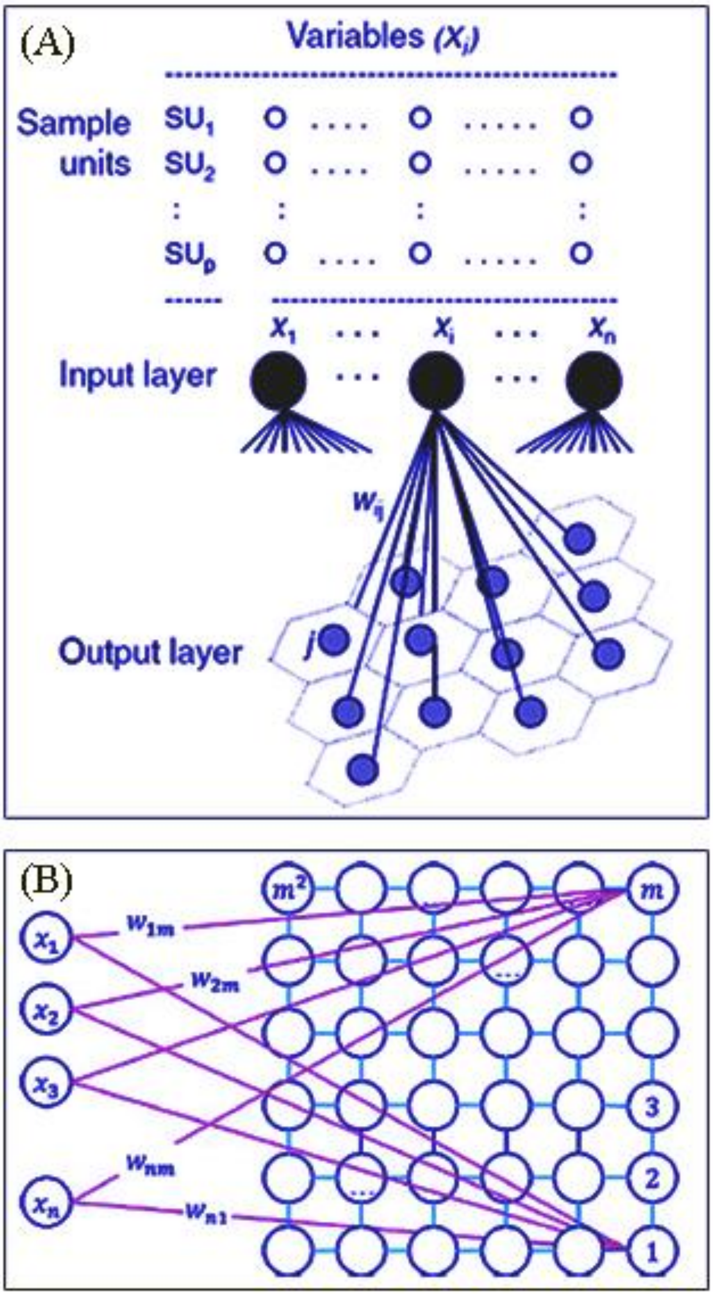

Figure S2. Schematic diagram (A) and basic structure (B) of the SOM network. The SOM network is an array of m^2 processing neurons. If these m neurons are arranged on a grid in a plane, the network is called two-dimensional because this network maps high-dimensional input vectors onto a two-dimensional surface. For a given network, the input vector x has a fixed dimension n. The n components of the input vector x (*i.e.*, x_1, x_2, . . ., x_n) are connected to each neuron in the array. A synaptic weight w_{ij} is defined for a connection from the i_{th} component of the input vector to the j_{th} neuron. Thus, an n-dimensional vector w_j of synaptic weights is associated with each neuron j j[1−3].

Table S1. Coding rules of 12 categories (46 characteristics) for the phylogenetic reconstruction. ‘0’ represents the original character state, ‘1’ or ‘2’ represent the derived character state, and ‘?’ represents a missing feature. ‘1’ represents more original characteristics than ‘2’, but ‘0’ represents more original characteristics than ‘1’.

No.	Categories	Characteristics	Acronym	Encoding rules
1	Stem	Type of underground stem	TUS	Sympodial-clumping (0); Sympodial-scattering (1)
2	Leaf sheath	Number	LSN	?
		Length relative to inflorescences	CPI	Longer (0); Shorter (1); Equal (2)
		Leaf present or absent	WOL	Absent (0); Present (1)
		Extent of expansion around the spathe	ES	Not expanded (0); Expanded slightly (1); Expanded (2)
3	Inflorescences	Genuine or false inflorescence	IGF	False (0); Genuine (1)
		Botryose or conical	IB/IC	Botryose (0); Conical (1)
		Compact or squarrose	IC/IS	Compact (0); Squarrose (1)
		Terminal or lateral	IL/IT	Terminal (0); Lateral (1)
		Bracts present or absent	IWOB	Absent (0); Present (1)
		Length (mm)	ILG	?
4	Spikelet	Number	SN	?
		Length (mm)	SLG	?
		Number of florets	SFN	?
		Color of florets	SFC	Light yellow green (0); Light green (1); Green (2)
		Length of rachilla (mm)	SRLG	?
		Length of pedicel (mm)	SPLG	?
		Hairs on pedicel present or absent	SPH	Absent (0); Present (1)
		Bracts at the base of pedicel	SPB	Absent (0); Present (1)
5	Glume texture	Glume and texture	GM	Papery (0); Membranous (1)
6	First glume	Shape	FS	Linear-lanceolate (0); Ovate-lanceolate (1); Long and narrow lanceolate (2)
		Length (mm)	FLG	?
		Number of nerves	FNN	?
7	Second glume	Shape	SS	Linear-lanceolate (0); Ovate-lanceolate (1); Long and narrow lanceolate (2)
		Length (mm)	SLG	?
		Number of nerves	SNN	?
8	Lemma	Shape	LS	Linear-lanceolate (0); Ovate-lanceolate (1); Long and narrow lanceolate (2)
		Length (mm)	LLG	?
		Number of nerves	LNN	?
		Hairs present or absent	LWOH	Absent (0); Present (1)
		Texture	LM	Papery (0); Membranous (1)
9	Palea	Length (cm)	PLG	?
		Two cristae present or absent	PWOC	Absent (0); Present (1)
		Apex split into two	PAS	No (0); Yes (1)
		Hairs on cristae present or absent	PCH	Absent (0); Present (1)

		Hairs between cristae	PBH	Absent (0); Present (1)
10	Lodicule	Number	LN	?
		Shape	LS	Lanceolate (0); Triangular (1); Ovate (2)
		Comparative size	LSSN	Equal (0); Not equal (1)
		Hairs on margins present or absent	LMH	Absent (0); Present (1)
11	Stamen	Number	SUN	?
		Color of anther	AAC	Yellow (0); Brownish yellow (1); Purple (2)
12	Gynoecium	Shape of ovary	GOS	Elliptic (0); Ovate (1); Oblong (2)
		Hairs present or absent	GWOH	Absent (0); Present (1)
		Number of styles	GSN	?
		Number of stigmas	GSTN	?

Figure S2. Coding matrix of the studied species according to the encoding rules. ‘0’ represents the original character state, ‘1’ or ‘2’ represent the derived character state, and ‘?’ represents a missing feature. ‘1’ represents more original characteristics than ‘2’, but ‘0’ represents more original characteristics than ‘1’.

Species	Acronym	Coding matrix
F. adpressa T.P. Yi	F.a	001111211102001117?020112010001200021112010?022120111
F. brevissima T.P. Yi	F.ba	00111121001110001011 2?11001011200120000021001010
F. denudata T.P. Yi	F.d	11112100102000100000000000000000000000000000000000
F. dracocephala T.P. Yi	F.dr	00111121001?201011120?12011011100011101100000 1001010
F. ferax (Keng f.) T.P. Yi	F.f	1111111111110100000000000000000000000000000000000
F. fungosa T.P. Yi	F.fu	1111211101102210000000000000000000000000000000000
F. grossa T.P. Yi	F.g	00111001110?000110110112010011000011100110002112210 1
F. hsuchiana T.P. Yi	F.h	0011111111011022 1?010022110001010011111?10?022020011
F. lincangensis T.P. Yi	F.l	00111001110?00011011011200100100001111011002102101 0
F. mairei (Hack. ex Hand.-Mazz) T.P. Yi	F.m	0011100111?1?1011?220112?01?01210010111?100121022000
F. melanostachys (Hand.-Mazz) T.P. Yi	F.me	111111111110011000000000000000000000000000000000
F. nitida (Mitford ex Stapf) Keng f.	F.n	0011112100112011110001110110111000100121 10?121122010
F. ostrina T.P.Yi	F.b	1111210002100210000000000000000000000000000000000
F. pauciflora (Keng f.) T.P. Yi	F.p	0011112100111001102101120000112002111101 0?122020101
F. robusta T.P. Yi	F.r	1111210011200010000000000000000000000000000000000
F. spathacea Franch.	F.sp	1111210012201110000000000000000000000000000000000
F. scabrida T.P. Yi	F.sc	0011101111011111?0212211101110001111121001020100100
T. aristatus E.G. Camus	T.a	0010002000011??1002?02?2?0??01100011102?000012022010
T. unispiculatus T.P. Yi & J.Y. Shi	T.u	10012000001000000000000000000000000000000000000000

Methods S1. The SOM is a sheet like network structure, in which a squared array of output units is commonly adopted. The array of the output units is called an output or feature map[4]. After completing training of a SOM net, the input vectors used for training will be mapped onto different output units. If two input vectors are mapped onto the same output unit, it means that these two input vectors have similar characteristics[3,5]. In the work, the algorithm is as follows[1,4,6]:

Step #1 Set up the map size and initialize the reference vectors $\mathbf{m}_j$ associated with each output node to random values.

Step #2 Present one input vector $\mathbf{x}(t)$.

Step #3 Calculate the distance d_j between the input vector $\mathbf{x}(t)$ and each output node j.

Step #4 Select the winner output node j that is associated with the reference vector $\mathbf{m}_j(t)$ that minimizes d_j.

Step #5 Update reference vectors for node j and its neighborhood by the function.

Step #6 Return to step 2 and repeat the cycle until convergence (see Figure 2).

$$\mathbf{m}_j(t + 1)=\mathbf{m}_\mathrm{j}(t) + a(t)\ h_{cj}(t)[\mathbf{x}(t) - \mathbf{m}_j(t)] \qquad (1)$$

Where $a(t)$ is the learning rate and h_{cj} is a neighborhood kernel. $a(t)$ is a decreasing function that controls the magnitude of the changes with the time [$0< a(t) <1$]. The neighborhood kernel is a function that determines the area of effect the input vector has on the map, and it is greatest for the winner neuron j, the reference vector that is closest to the input vector, and monotonically decreases the farther away neuron i is from neuron j on the map grid.

References

1. Kohonen, T. Self-organized formation of topologically correct feature maps. *Biol. Cybern.* **43**, 59–69, doi: 10.1007/BF00337288 (1982).
2. Céréghino, R., Santoul, F., Compin, A., & Mastrorillo, S. Using self-organizing maps to investigate spatial patterns of non-native species. *Biol. Conserv.* **125**, 459–465, doi:10.1016/j.biocon.2005.04.018 (2005).
3. Ghaseminezhad, M.H., & Karami, A. A novel self-organizing map (SOM) neural network for discrete groups of data clustering. *Appl. Soft Comput.* **11**, 3771–3778, doi: 10.1016/j.asoc.2011.02.009 (2011).
4. Vesanto, J. SOM-based data visualization methods. *Intelligent Data Anal.* **3**, 111–126, doi: 10.3233/IDA-1999-3203 (1999).
5. Kandel, E.R., Schwartz, J.H., & Jessell, T.M. *Principles of Neural Science*, 4th ed., The University of Chicago Press, Chicago, IL, 231–232 (2000).
6. Kohonen, T. Essentials of the self-organizing map. *Neural Networks* **37**, 52–65, doi: 10.1016/j.neunet.2012.09.018 (2013).
